# Curcuminoid-Tailored Interfacial Free Energy of Hydrophobic Fibers for Enhanced Biological Properties

**DOI:** 10.1021/acsami.1c05034

**Published:** 2021-05-24

**Authors:** Wevernilson F. de Deus, Bruna M. de França, Josué Sebastian B. Forero, Alessandro E. C. Granato, Henning Ulrich, Anelise C. O. C. Dória, Marcello M. Amaral, Adam Slabon, Bruno V. M. Rodrigues

**Affiliations:** † Instituto Científico e Tecnológico, Universidade Brasil, Rua Carolina Fonseca 235, 08230-030, São Paulo, São Paulo, Brazil; ‡ Instituto de Química, Universidade Federal do Rio de Janeiro, Centro de Tecnologia, Bloco A, Cidade Universitária, 21941-909, Rio de Janeiro, Rio de Janeiro, Brazil; § Departamento de Bioquímica, Instituto de Química, Universidade de São Paulo, CEP 05508-000, São Paulo, São Paulo, Brazil; ∥ Laboratório de Biotecnologia e Plasmas Elétricos, IP&D, Universidade do Vale do Paraíba, Avenido Shishima Hifumi 2911, 12244-000, São José dos Campos, São Paulo, Brazil; ⊥ Department of Materials and Environmental Chemistry, Stockholm University, Svante Arrhenius väg 16C, 10691 Stockholm, Sweden

**Keywords:** electrospinning, poly(lactic acid), curcuminoids, SH-SY5Y cells, surface free energy, cell adhesion, cell proliferation

## Abstract

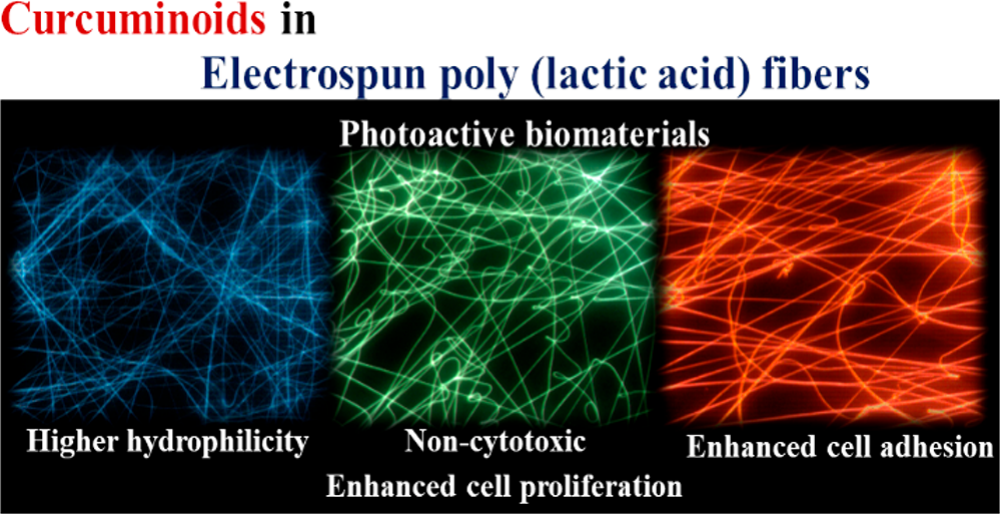

The ability of mimicking the extracellular matrix architecture has gained electrospun scaffolds a prominent space into the tissue engineering field. The high surface-to-volume aspect ratio of nanofibers increases their bioactivity while enhancing the bonding strength with the host tissue. Over the years, numerous polyesters, such as poly(lactic acid) (PLA), have been consolidated as excellent matrices for biomedical applications. However, this class of polymers usually has a high hydrophobic character, which limits cell attachment and proliferation, and therefore decreases biological interactions. In this way, functionalization of polyester-based materials is often performed in order to modify their interfacial free energy and achieve more hydrophilic surfaces. Herein, we report the preparation, characterization, and *in vitro* assessment of electrospun PLA fibers with low contents (0.1 wt %) of different curcuminoids featuring π-conjugated systems, and a central β-diketone unit, including curcumin itself. We evaluated the potential of these materials for photochemical and biomedical purposes. For this, we investigated their optical properties, water contact angle, and surface features while assessing their *in vitro* behavior using SH-SY5Y cells. Our results demonstrate the successful generation of homogeneous and defect-free fluorescent fibers, which are noncytotoxic, exhibit enhanced hydrophilicity, and as such greater cell adhesion and proliferation toward neuroblastoma cells. The unexpected tailoring of the scaffolds’ interfacial free energy has been associated with the strong interactions between the PLA hydrophobic sites and the nonpolar groups from curcuminoids, which indicate its role for releasing hydrophilic sites from both parts. This investigation reveals a straightforward approach to produce photoluminescent 3D-scaffolds with enhanced biological properties by using a polymer that is essentially hydrophobic combined with the low contents of photoactive and multifunctional curcuminoids

## Introduction

The self-healing potential of many tissues in the human body is unfortunately limited. In this context, polymeric scaffolds have been widely used as coadjutant materials in order to assist and/or accelerate the process of tissue regeneration.^1,2^ To meet the requirements for the engineering of living tissues, a material should ideally mimic the structural architecture and components of the extracellular matrix (ECM). Thus, an ideal scaffold must present not only suitable mechanical properties but also a good environment for cell seeding, adhesion, and proliferation.^3^ Polymeric scaffolds have gained much attention over the last two decades.^2,4,5^ In this context, the fabrication of polymeric architectures aiming at the native tissues has been one of the main challenges in the field. For this purpose, several methods have been developed and refined to generate nanoscale scaffolds as ECM substitutes, such as molding,^6^ microfluidics,^7^ phase separation,^8^ drawing,^9^ and electrospinning.^4,10,11^ Among these methods, electrospinning has been consolidated as a simple, versatile and cost-effective technique to produce ultrathin and nanofibers from a wide range of polymers.^12,13^ Because of its intrinsic high porosity and interconnectivity, electrospun fibers have been the target of many applications in the biomedical field, including drug delivery,^14,15^ tissue engineering,^10,11,14^ and wound dressing.^16−18^

The high surface-to-volume ratio of electrospun fibers increases their bioactivity while enhancing the bonding strength with the host tissue.^3,10^ In addition, due to the ability of mimicking the ECM nanofilamentary architecture, electrospun fibers provide 3D environments that enhance cell-scaffold and cell–cell interactions.^3,19^ Thus, cell-frameworks based on EMC-like electrospun scaffolds have been extensively studied due to their great capacity to promote cell adhesion, proliferation, and tissue formation.^3,19^

Many biocompatible polyesters have found a prominent place in biomedical applications due to their easy processability. Among numerous examples, we can highlight the poly(lactic acid) (PLA),^15,20,21^ poly(butylene adipate-*co*-terephthalate) (PBAT),^22,23^ and polycaprolactone (PCL)^24^ due to a unique combination of their properties, including renewability, biocompatibility,, and relatively low cost. However, one can say that special attention has been directed to PLA over the usual polymers/polyesters for biomedical applications. While the U.S. Food and Drug Administration (FDA) has approved its use and contact with biological human fluids since 1970, this polymer represents also an excellent choice for energy saving. PLA degradation generates CO_2_ and H_2_O as subproducts; while both are obviously not hazardous, the combination of all its aforementioned properties makes this polyester an ideal candidate for tissue engineering.^25,26^ However, as other polyesters, PLA presents a high hydrophobic character, limiting cell attachment and proliferation and, therefore, decreases biological interactions. In this way, surface functionalization/modification of polymeric materials has been often performed to overcome these drawbacks.^27,28^ This should be seen not only as a strategy to improve the scaffold functionality into the regenerative medicine field but many times also as a mandatory step to create a successful final material.

Curcumin (CUR) is a well-known yellow-colored polyphenol, which is an active ingredient of turmeric. To date, investigations have been reporting the antioxidant, antibacterial, antiviral, anticancer, and anti-inflammatory properties of this compound into many different approaches.^29^ Furthermore, this polyphenol has shown outstanding *in vitro* and *in vivo* results in biomedical studies involving different diseases, such as diabetes.^29^ Recent investigations have demonstrated the effectiveness of CUR in diminishing the oxidative damage associated with aging and also to treat brain ischemia^30^ and Alzheimer’s disease.^31^ It has been also highlighted the ability of CUR to improve the proliferation of stem cells,^32^ which is crucial for the process of tissue regeneration.

Curcuminoids, which has curcumin as the main compound, are molecules that feature a π-conjugated system and a central β-diketone unit. These molecules display a π-conjugated D–A–D structure, in which A and D represent electron acceptor and donor groups, respectively. These compounds comprise a class of photo- and electroactive molecules, which find a wide range of applications in photovoltaics,^33^ (bio)-imaging,^34^ organic electronics^35^ and other fields. The optical and electronic properties of these compounds have been already extensively investigated elsewhere.^33,36^ It has been reported that the electron donor character of one terminal D strongly influences the reduction potential due to the strong resonance interaction along the molecule backbone.

Although the literature reveals numerous investigations on (electrospun) hybrid materials containing curcumin for a wide range of applications, the major part has been focusing on high curcumin contents (usually from 2 to 10 wt %) into polymer matrices aiming at its controlled release for antibacterial, antioxidant, and anti-inflammatory purposes. Nevertheless, up to date there is no study investigating whether small contents of curcumin and other related-curcuminoids influence the interfacial free energy of electrospun matrices with respect to optical application and interaction with biological matter.

**Figure 1:**
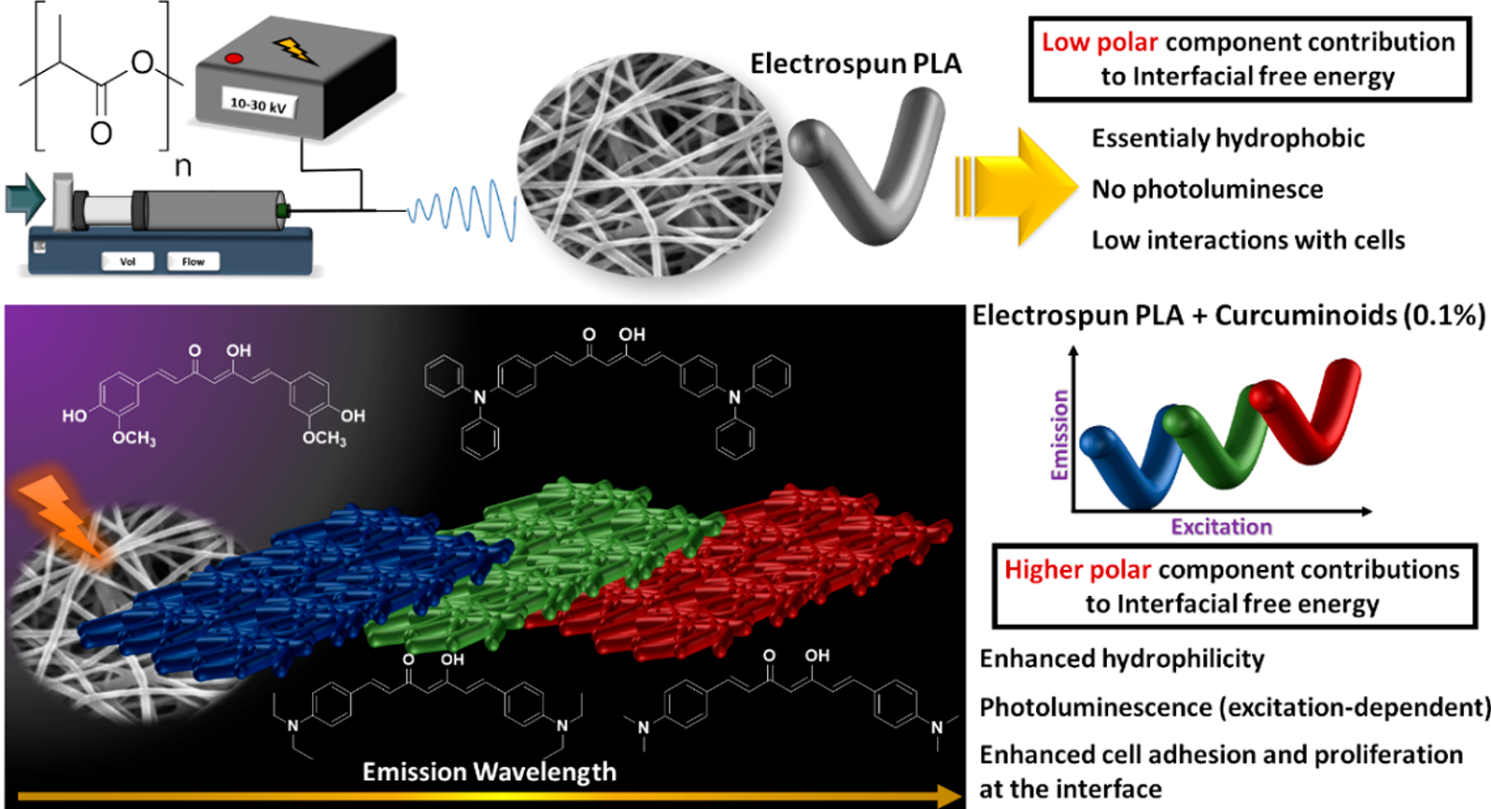
Schematic representation of the combination of PLA and curcuminoids in order to reach hybrid materials with enhanced photochemical and biological properties.

Herein, we report the preparation, characterization, and *in vitro* assessment of electrospun polyester fibers containing low contents of different curcuminoids (Figure 1). While investigating the achievement of functional, homogeneous, and defect-free fibrous architectures by combining PLA and low contents of curcuminoids, we evaluated the potential of these materials into future biomedical applications. For this purpose, we investigated their optical properties, water contact angle, and surface properties while assessing their *in vitro* behavior using SH-SY5Y cells.

## Experimental Section

### Physical Measurements

Reagent quality solvents were obtained from TEDIA and used as supplied unless otherwise stated. Melting points were determined using a Thomas Model 40 Micro Hot Stage (Kofler-type) melting point apparatus and were given uncorrected. Infrared spectra were recorded on a Nicolet Magna IR-FT spectrophotometer and examined as KBr pellets (0.01%). ^1^H- and ^13^C NMR (Bruker DPX-500) spectra were recorded in DMSO-*d*_6_ and CDCl_3_ at 400 and 125 MHz, respectively. Spectroscopy measurements were carried out with spectroscopic grade solvents (HPLC). The UV–visible absorption spectra were recorded using a Shimadzu UV-2425 spectrophotometer with 1 cm quartz cuvettes. Fluorescence measurements were obtained at room temperature using a steady-state/time-resolved spectrofluorometer Edinburgh Instruments FLS 900.

### General Synthetic Method for Curcuminoids

The synthesis of curcumin derivatives was performed according to a literature method^37,38^ with modifications. Acetyl acetone (10 mmol) and boric anhydride (5.0 mmol) were dissolved in ethyl acetate (30 mL) and stirred at 80 °C for 1 h. The appropriate benzaldehyde (4-hydroxy-3-methoxybenzaldehyde; 4-(diphenylamino)benzaldehyde; 4-(dimethylamino)benzaldehyde; and 4 (diethylamino)benzaldehyde)) (20 mmol) dissolved in ethyl acetate and tributyl borate (40 mmol) was added. The reaction was stirred at 80 °C for 1 h; then *n*-butylamine (5.0 mmol) in ethyl acetate (5 mL) was added dropwise over 15 min, and the mixture was stirred for 4 h at 80 °C. The progress of the reaction was monitored by TLC analysis. Upon complete consumption of the starting material (as indicated by TLC), hydrochloric acid (0.25 M) was added and the mixture was stirred for 1 h. Organic layers of water-immiscible solvents were separated and extracted three times with ethyl acetate. The combined organic layer was washed with water and brine, dried over Na_2_SO_4_, filtered through a pad of silica, and concentrated under reduced pressure, leading to crude powder, which was further purified by flash chromatography (silica gel, hexane/ethyl acetate, 2:1, v/v).

#### 5-hydroxy-1,7-bis(4-hydroxy-3-methoxyphenyl)hepta-1,4,6-trien-3-one (Curcumin, named CUR)

Orange crystals (yield 60%) from water/ethanol. mp = 180 °C.^39^ IR (ν_max_/cm^–1^) 3431.54 (O–H) 3014.86, 2937.69 (C–H aromatic/aliphatic), 2841.24 (O–CH_3_), 1629.76 (C=C), 1589.25 (C=O), 1512.09 (C=C_ar_). ^1^H NMR (CDCl_3_, 400 MHz): δ (ppm) 9.71 (s, 2H), 8.28 (br, 1H enol form), 7.55 (d, *J* = 16.42 Hz, 2H), 7.15 (d, *J* = 8.56 Hz, 2H), 6.83 (d, *J* = 8.04 Hz, 2H), 6.76 (d, *J* = 15.81 Hz, 2H), 6.06 (br, 1H enol form) 3.83 (s, 6 H, O–CH_3_). ^13^C NMR (CDCl_3_, 125 MHz): δ (ppm) 183.67 (C=O), 149.79 (C–OH), 148.47 (C–OMe), 141.17, 126.82, 123.57, 121.56, 116.18, 111.78, 101.33, 56.16 (O–CH_3_).

#### 1,7-Bis(4-(diphenylamino)phenyl)-5-hydroxyhepta-1,4,6-trien-3-one (−N(Ph)_2_)

Red crystals (yield 30%) from hexane/ethyl acetate. mp = 240 °C.^40^ IR (ν_max_/cm^–1^) 3056.67, 3025.81 (C–H aromatic), 1625.72 (C=C), 1585.23 (C=O), 1504.23, 1488.80 (C=C_ar_), 1322.95 (C_ar_–N). ^1^H NMR (CDCl_3_, 400 MHz): δ (ppm) 16.68 (br, 1H, OHC=CH), 8.15 (d, *J* = 15.82 Hz, 2H), 7.95 (d, *J* = 8.63 Hz, 4H), 7.84 (t, *J* = 7.54 Hz, 8H), 7.69–7.62 (m, 12H), 7.56 (d, *J* = 8.84 Hz, 4H), 7.04 (d, *J* = 15.85 Hz, 2H), 6.33 (s, 1H).

#### 1,7-Bis(4-(dimethylamino)phenyl)-5-hydroxyhepta-1,4,6-trien-3-one (−N(CH_3_)_2_)

Purple powder (yield 40%). mp = 205 °C.^37^ IR (ν_max_/cm^–1^) 3033.53, 2917.82, 2850.32 (C–H aromatic/aliphatic), 1591.01 (C=C), 1525.44 (C=O), 1479.16 (C=C_ar_), 1363.45 (C_ar_–N), 1186.03 (C–N aliphatic). ^1^H NMR (DMSO-*d*_6_, 400 MHz): δ (ppm) 7.54 (d, *J* = 8.05 Hz, 6H), 6.74 (d, *J* = 8.64 Hz, 2H), 6.62 (d, *J* = 15.64 Hz, 2H), 5.96 (s, 2H), 2.99 (s, 12H).

#### 1,7-Bis(4-(diethylamino)phenyl)-5-hydroxyhepta-1,4,6-trien-3-one (-N(C_2_H_5_)_2_)

Dark red crystals (yield 40%) from isopropyl alcohol. mp = 145 °C.^41^ IR (ν_max_/cm^–1^) 2971.81, 2929.39 (C–H aliphatic/aromatic), 1589.08 (C=O), 1519.66 (C=C), 1353.81 (C_ar_–N), 1184.10 (C–N aliphatic). ^1^H NMR (CDCl_3_, 400 MHz): δ (ppm) 16.43 (br, 1H), 7.60, 6.41 (dd, *J* = 15.8 Hz, each 2H), 7.44 (d, *J* = 9.63 Hz, 4H), 6.65 (d, *J* = 8.4 Hz, 4H), 5.73 (s, 1H), 3.42 (q, *J* = 6.96 Hz, 8H), 1.23 (t, *J* = 7.26 Hz, 12H); ^13^C NMR (CDCl_3_, 125 MHz): δ (ppm) 183.42 (C=O), 149.28, 140.67, 130.23, 122.36, 118.67, 111.46, 100.84, 44.58 (N(CH_2_CH_3_)_2_), 12.73 (N(CH_2_CH_3_)_2_).

### Electrospinning of PLA and PLA/Curcuminoids Solutions

Pure PLA (Ingeo, Biopolymer 200 from NatureWorks LLC) solutions were prepared at a concentration of 10% (w/v) using 1 g of the polymer pellets. Initially, the polymer was added to 7.5 mL of chloroform (99%, Sigma-Aldrich) in a flask, which was kept under magnetic stirring at room temperature for 3 h until full dissolution. Next, 2.5 mL of DMF (99.8%, Sigma-Aldrich) was added, and the resulting solution was kept under stirring for additional 30 min. Solutions containing curcuminoids were prepared using the aforementioned procedure plus an additional step: 1 mg of each compound was added to the resulting solutions, aiming at final solutions with low curcuminoid contents (0.1% wt %). The final PLA/curcuminoid solutions were stirred for 30 min until homogenization. All solutions were fully dissolved with no remaining particles.

Electrospinning was carried out using a high-voltage source (Faíscas, Model FD + 30 kV) operating at 10 kV for PLA solutions and 18 kV for PLA/curcuminoid solutions. All samples were electrospun using 23 G metallic needles and 10 cm as needle-collector distance. A square copper plate (6.0 cm^2^) covered with aluminum foil was used as the negative electrode to collect the fibers. A vertical system was set in order to provide slow flow of the solution by gravity. Each electrospinning experiment was performed for 60 min, and the temperature and humidity were carefully controlled in the ranges of 28–30 °C and 30–35%, respectively.

Table 1 summarizes all the electrospun samples prepared in this investigation and their respective codes used throughout the text.

**Table 1:** Electrospun Samples and Their Respective Codes

sample	polymer	curcuminoid	electrospun sample code
control	PLA		PLA
1	PLA	CUR	PLA–CUR
2	PLA	N(CH_3_)_2_	PLA–N(CH_3_)_2_
3	PLA	N(C_2_H_5_)_2_	PLA–N(C_2_H_5_)_2_
4	PLA	N(Ph)_2_	PLA–N(Ph)_2_

### Characterization of Electrospun PLA and PLA/Curcuminoids

A Shimadzu RF-6000 spectrophotometer was used to perform the fluorescence measurements of all electrospun mats. Photomicrographs were taken using a fluorescence microscope (Leica Epifluorescence Microscope DMLB with a camera to capture pictures; model Leica DFC310FX, Nussloch, Germany). Field emission gun scanning electron microscopy (FEG-SEM) was carried out using a JEOL Microscope (model JSM 7401F). All samples were previously coated with a thin layer of gold (∼19 nm) using a sputter coater. Attenuated total reflectance Fourier transform infrared spectroscopy (ATR-FTIR) was carried out in a PerkinElmer Spotlight 400 FTIR Imaging System, and data were collected in the range of 4000–400 cm^–1^ in transmittance mode.

For each sample, the contact angle (θ) was measured by using the sessile drop method. For this, the liquid of interest was automatically dropped using a computer-controlled system attached to a Krüss EasyDrop contact angle instrument (EasyDrop DSA 100). In order to calculate the surface energy, distilled water and diiodomethane were used, according to Owens method.^42^ The equipment calibration was performed using CP23 and CP24 sets, which contained standards that accurately followed the theoretical drop shape according to Young–Laplace. Measurements for each sample were carried out in triplicate (different regions) in a controlled humidified atmosphere.

The surface energy, composed by polar and dispersive components, was calculated using the measured contact angles via the interfacial tension through the Young’s (eq 1)^43^ and Young–Dupré (eq 2) equations
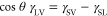
1
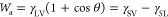
2

In these equations, θ value refers to the contact angle between the liquid and the solid, γ_LV_, γ_SV_, and γ_SL_ are the interfacial energies between the liquid/vapor, solid/vapor, and solid/liquid interfaces, and *W*_α_ is the adhesion energy per unit area of the solid and liquid surfaces. Equation 1 and eq 2 can be combined into eq 3, often called Owens, Wendt, Rabel, and Kaelble (OWRK) equation^44^
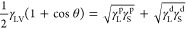
3

In eq 3, the terms γ_L_^p^ and γ_S_^p^ refer to the polar components of the surface energy from the liquid and solid phases, respectively; γ_L_^d^and γ_S_^d^ are related to the dispersive components of the surface energy from the liquid and solid phases, respectively. While γ_L_^d^ and γ_L_^p^ values are reported in the literature for many liquids, γ_S_^d^ and γ_S_^p^ can be calculated by an approximation using a single measurement of contact angle with eq 3.^44^ Herein, we discriminated the polar and dispersive components of the surface energy of our materials by measuring the contact angles using water and diiodomethane, which are liquids with well-known and reported polar and dispersive components of surface energy.^45^

### Cytotoxicity Assay

SH-SY5Y cells were cultured according to the protocol recommended by the ATCC (American Type Culture Collection). Briefly, the cells were grown in a 75 cm^2^ culture flask with 15 mL of compound culture medium by DMEM with 10% fetal bovine serum (FBS; Cultilab), 1% l-glutamine (Invitrogen), and 1% penicillin/1% streptomycin (Gibco-BRL). The cells were maintained at 37 °C and 5% CO_2_. In 60% of confluence, cells were trypsinized at a ratio of 1:5. Cell viability was assessed by MTT assay (3- (4, 5-dimethylthiazolyl-2)-2,5-diphenyltetrazolium). The cells were seeded in 24 well plates for 24 h and maintained at 37 °C and 5% CO_2_ to allow attachment. After this period, electrospun samples previously UV sterilized (1 × 1 cm) were put inside the wells. Cells were incubated with the samples for 24 and 120 h at 37 °C and 5% CO_2_. Next, the culture medium was removed and 300 μL of MTT solution (0.5 mg/L) was added to each well and incubated for 3 h at 37 °C. Then, MTT was aspirated and the reaction product, formazan salt, was dissolved by adding 400 μL of dimethyl sulfoxide (DMSO, Sigma-Aldrich) in each well. The plate was then shaken for 15 min and the content transferred to a 96-well plate. Next, the optical density was read at 540 nm on an ELISA plate reader (Labsystems Multiskan, MS, U.S.A.).

### Scanning Electron Microscopy

Electrospun samples seeded with neuro2a cells were fixed in 2.5% glutaraldehyde solution buffered in a 0.1 M sodium cacodylate solution, pH 7.2. After the fixation process, samples were washed with 0.1 M sodium cacodylate buffer, pH 7.2, and submitted to a process of metallic impregnation. To make this, samples were incubated in 2% osmium tetroxide in 0.1 M sodium cacodylate buffer, pH 7.2 for 2 h, washed with 0.1 M sodium cacodylate buffer pH 7.2 three times during 15 min, and incubated in 1% tannic acid water solution for 45 min, followed by two washes in distilled water (10 min each).

After metallic impregnation, samples were dehydrated gradually in 50–70–90% ethanol (twice, 30 min each) and 100% (three times, 30 min each), and then samples were submitted to a drying process in a critical point chamber (Balzers CPD 030, Lichtenstein) using CO_2_. Samples were then coated with a thin layer of 20–30 nm thickness of gold (Sputtering, Leica Microsystems, Germany) and scanned on a FEI Quanta 250 FEG scanning electron microscope (ThermoFisher, U.S.A.).

### Statistical Analysis

Results were expressed as mean ± standard deviation. Statistical analyses were carried out using the open-source statistical programming language R v.3.3.0. Data were tested for normality and homogeneity of variances using the Shapiro–Wilks test of normality and an F test. Statistical significance between different treatments was determined using analysis of variance (ANOVA), post hoc Tukey’s test; *p*-values ≤0.05 were used to determine significant differences.

## Results and Discussion

**Figure 2:**
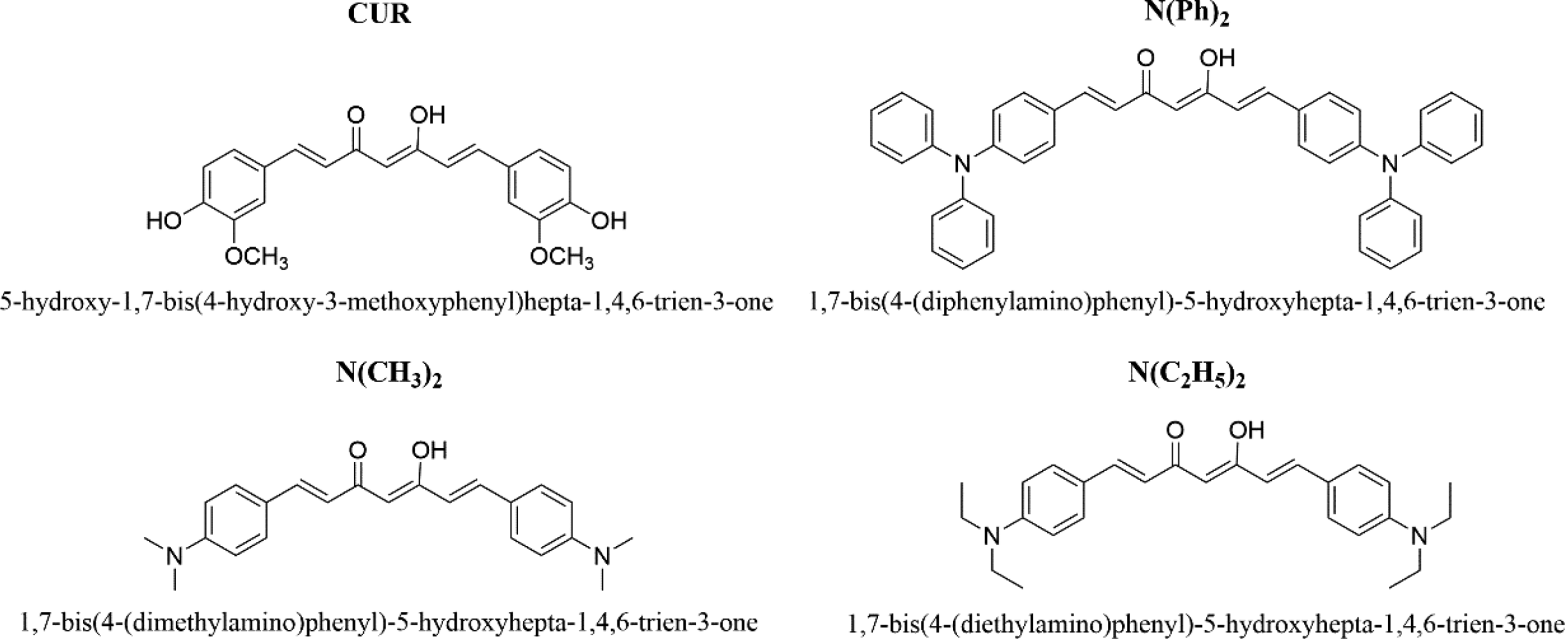
Chemical structures of all curcuminoids synthesized in this work.

In this investigation, different curcuminoids were synthesized based on previous works.^34,36,37^ These compounds contained π-conjugated D–A–D structures in which A and D represent electron acceptor and donor groups, respectively (Figure 2). Herein, the following curcuminoids were prepared: 5-hydroxy-1,7-bis(4-hydroxy-3-methoxyphenyl) hepta-1,4,6-trien-3-one (hereafter referred as CUR); 1,7-bis(4-(diphenylamino)phenyl)-5-hydroxyhepta-1,4,6-trien-3-one (hereafter referred as −N(Ph)_2_); 1,7-bis(4-(dimethylamino)phenyl)-5-hydroxyhepta-1,4,6-trien-3-one (hereafter referred as −N(CH_3_)_**2**_); 1,7-bis(4 (diethylamino)phenyl)-5-hydroxyhepta-1,4,6-trien-3-one (hereafter referred as −N(C_2_H_5_)_**2**_). The respective NMR spectra for each compound can be found in the Supporting Information.

**Figure 3:**
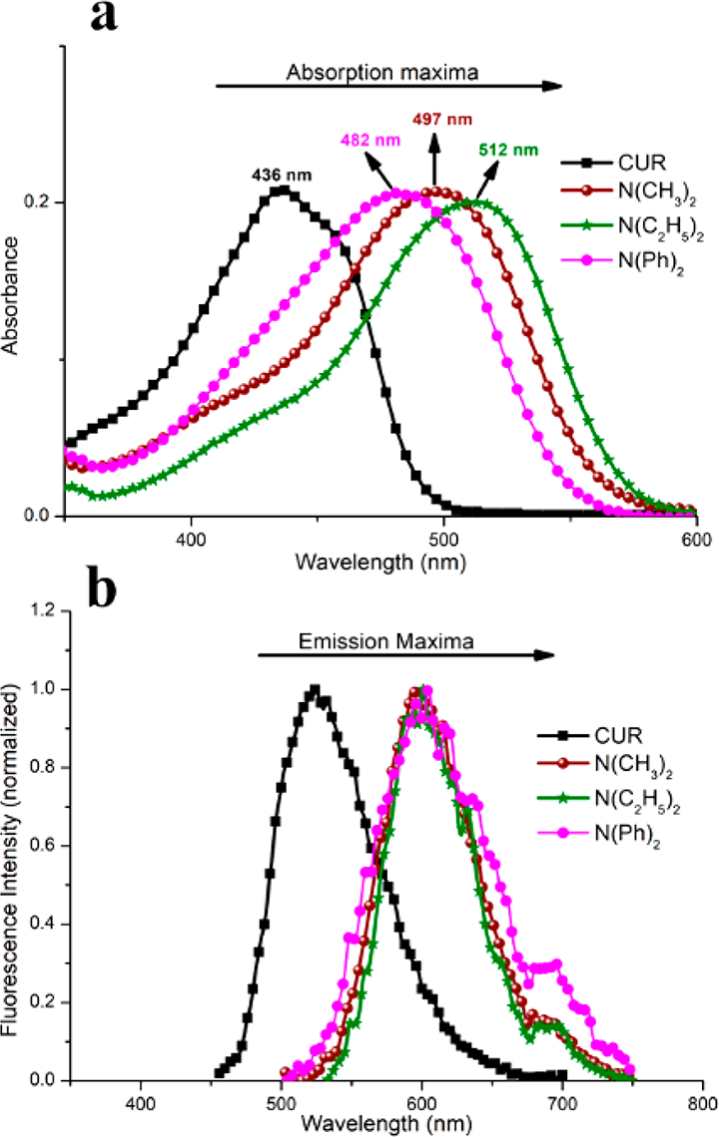
(a) Absorption spectra in the UV–vis region and (b) fluorescence emission spectra for curcuminoids in dimethyl sulfoxide.

Figure 3 shows the UV–vis and fluorescence spectra in DMSO solution for the curcuminoids used in the present study.

The curcumin molecule shows a strong UV–vis absorption, which is attributed to the π–π* transition of the carbonyl groups^46^ with the maximum emission (λ_max_) usually ranging in a broad interval between 400 and 440 nm depending on the solvent polarity, π-bonding nature, and hydrogen bond donating and accepting properties; all of these solvent properties influence deeply the excited state photophysics of curcumin.^47^ In the present work, using a polar solvent (DMSO), curcumin showed a maximum absorption (Figure 3a) at 436 nm. In nonpolar solvents, such as hexane, the curcumin absorption spectra are blue-shifted, while red-shifted in most hydrogen bond acceptor and proton donor solvents.^48,49^ As it can be observed from the absorption spectra (Figure 3a), all other curcuminoids presented a maximum absorption within the visible region, also attributed to the π–π* transitions of the carbonyl groups with a clear displacement to the red region due to the presence of different donor groups. A displacement in the order of 75 nm was observed between CUR and N(C_2_H_5_)_2_. This shift can be explained by the increase in the electron-donating character of the substituents introduced,^36^ which increases in the following order: 4-hydroxy-3-methoxyphenyl < 4-diphenylamine-phenyl < 4-dimethylamino-phenyl < 4-diethylamino-phenyl.

The curcumin fluorescence has also a strict dependence on the solvent polarity.^47,50^ While the maximal fluorescence in aprotic solvents, such as acetone and chloroform, is centered in the range of 494–538 nm, a red-shift to the region of 536–590 nm is observed for hydrogen bond donors like alcohols and DMF.^50^ For instance, nonpolar solvents present blue-shifts to the region of 445–470 nm.^47,50^Figure 3b shows the fluorescence emission spectra for all curcuminoids in DMSO. A similar trend was observed with a shift toward the red region due to the introduction of substituents with different electron-donating characters. While a maximum emission at 523 nm was observed for curcumin, the other curcuminoids had a maximum in the range of 593–604 nm.

**Figure 4:**
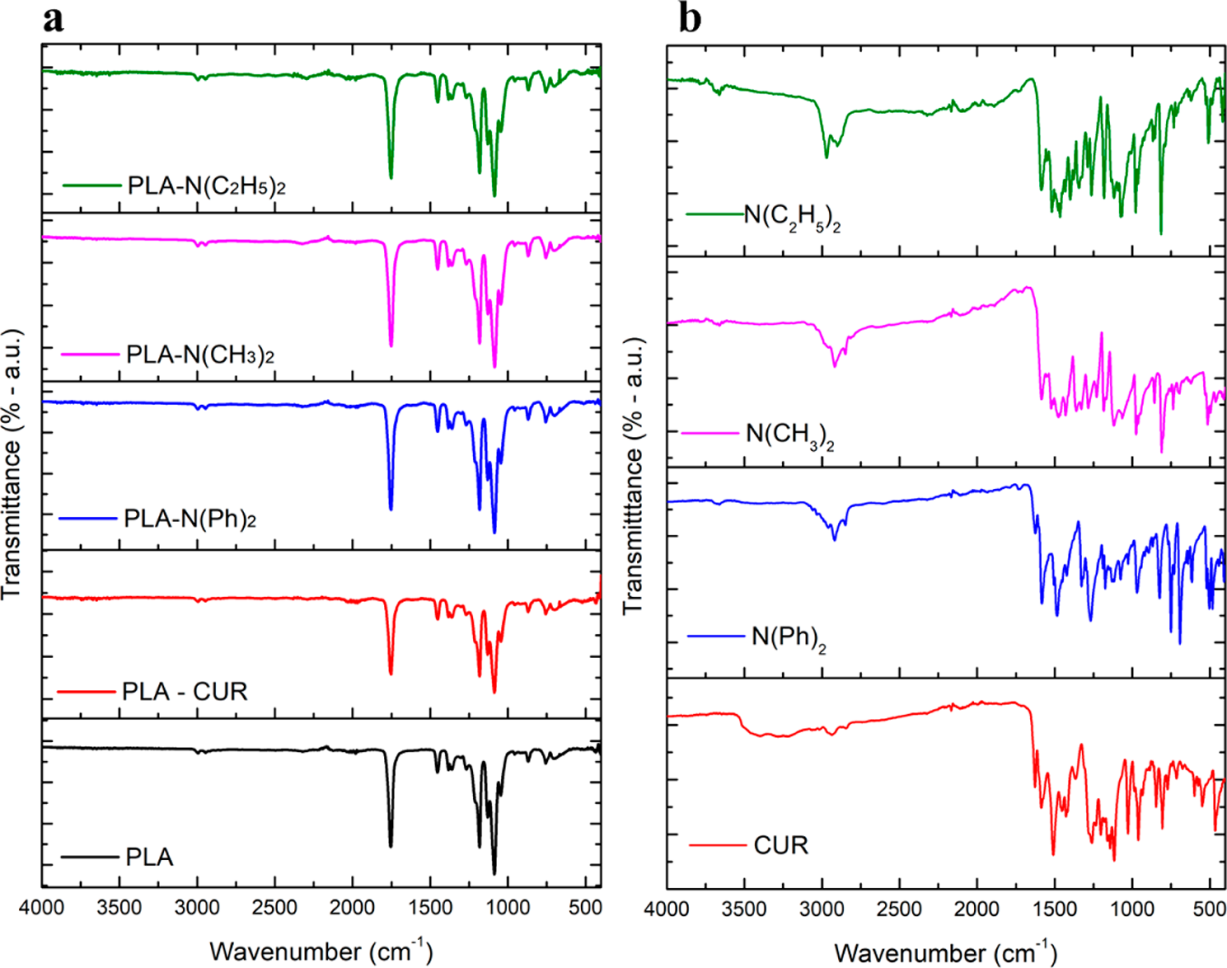
FTIR spectra for (a) electrospun PLA and electrospun PLA/curcuminoids and (b) as-prepared curcuminoids.

FTIR was used to obtain more in-depth information about the chemical structures, especially with regard to the chemical bonds. In line with other works in the literature, the PLA spectrum (Figure 4a) showed typical peaks located at 2995 and 2944 cm^–1^, which are attributed to the stretching vibrations of the CH_2_ groups. In the regions of 1753, 1183, 1130, and 1087 cm^–1^, typical high intensity and narrow bands were observed, which are related to the stretching of the −C–O and C–O–C bonds.^51^ The curcumin spectrum (Figure 4b) showed its signature peaks as follows:^52^ 3330 cm^–1^, stretching vibration from the −O–H groups; 3055 cm^–1^, stretching vibration from aromatic C–H bonds; 2955 cm^–1^, asymmetric vibration from −CH_3_– groups; 2930 cm^–1^, −CH_2_ asymmetric stretching; 1627 cm^–1^, stretching from the C=O bond; 1587 cm^–1^, C=C bond vibration in aromatics; 1510 cm^–1^, vibration of angular deformation from the benzene ring; 1427 cm^–1^, angular deformation from the CH_2_ group; 1368 cm^–1^, angular deformation from the CH_3_ group; 1118 cm^–1^, stretching from the C–O bonds.

A great similarity between the spectra of curcumin and the other curcuminoids (Figure 4b) was observed, which would be indeed expected due to their structural similarities. Nevertheless, when comparing to curcumin, we can observe a clear decrease in absorption in the 3330 cm^–1^ region for the other curcuminoids, associated with the replacement of the −OH groups. Conversely, an increase in absorption in the region of 3060–2900 cm^–1^ is noticed due to the greater presence of aromatic and aliphatic C–H groups arising from the substitutions. Furthermore, an absorption band is observed at 1330 cm^–1^ for all curcuminoids except curcumin, which is related to the vibration of the C–N bond in aromatics.

As expected, the low content of curcuminoids (0.1%) into the electrospun PLA matrix did not promote noticeable changes in the FTIR spectra (Figure 4a). Evidently, the processing of the material in all its stages (solubilization, electrospinning, and drying) did not lead to structural changes in the matrix with the preservation of the polymer structure.

**Figure 5:**
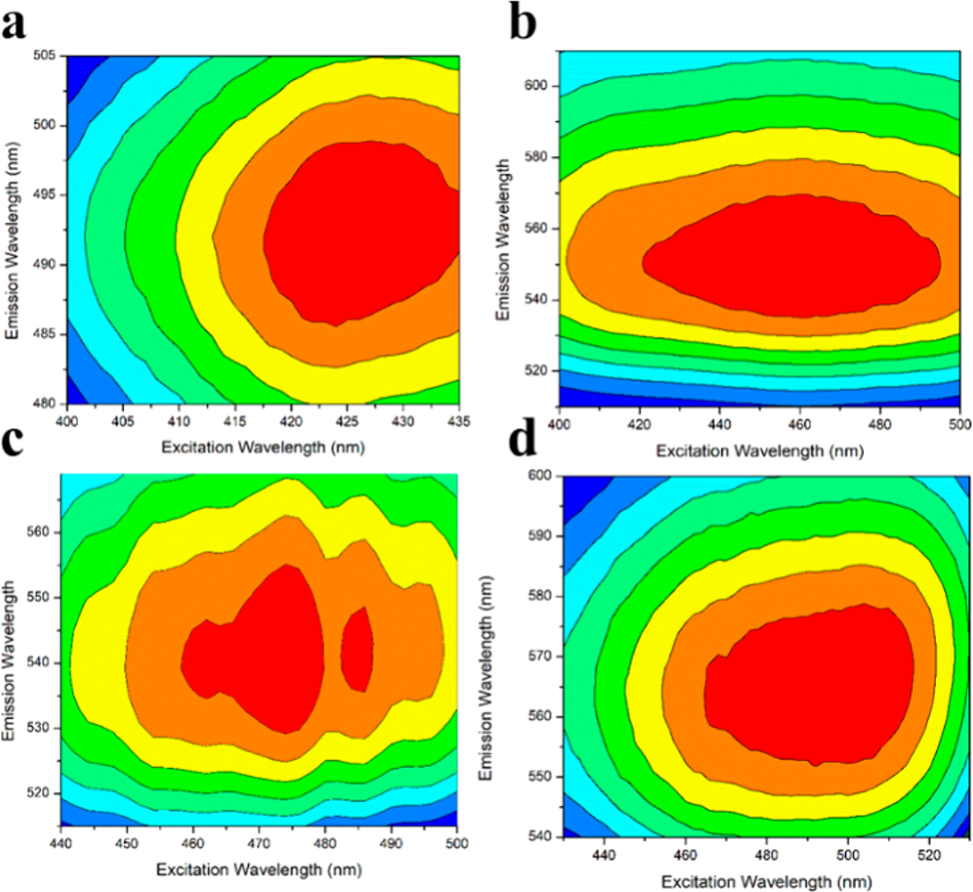
The 3D emission versus excitation maps for (a) PLA–CUR; (b) PLA–N(Ph)_2_; (c) PLA–N(CH_3_)_2_; and (d) PLA–N(C_2_H_5_)_2_

Figure 5 shows the 3D emission versus excitation maps for all electrospun PLA/curcuminoids mats. These three-dimensional maps represent a scan of these materials under different excitation wavelengths in the visible spectrum and the consequential fluorescence emission.

From the analysis of the 3D maps (Figure 5), it can be observed that all electrospun materials had a wide range of fluorescence emission, which was excitation-dependent. Analogously to the analysis of these compounds in liquid phase, the fluorescence does not arise from the polymeric matrix but due to the π–π* transitions of the carbonyl groups present in curcuminoids.^46^ The analysis of the 3D maps led to excitation maxima in the order of 427 nm for PLA–CUR, 460 nm for PLA–N(Ph)_2_, 470–484 nm for PLA–N(CH_3_)_2_, and 492 nm for PLA–N(C_2_H_5_)_2_. The emission maximums were of the order of 493, 552, 540, and 564 nm, respectively.

**Figure 6:**
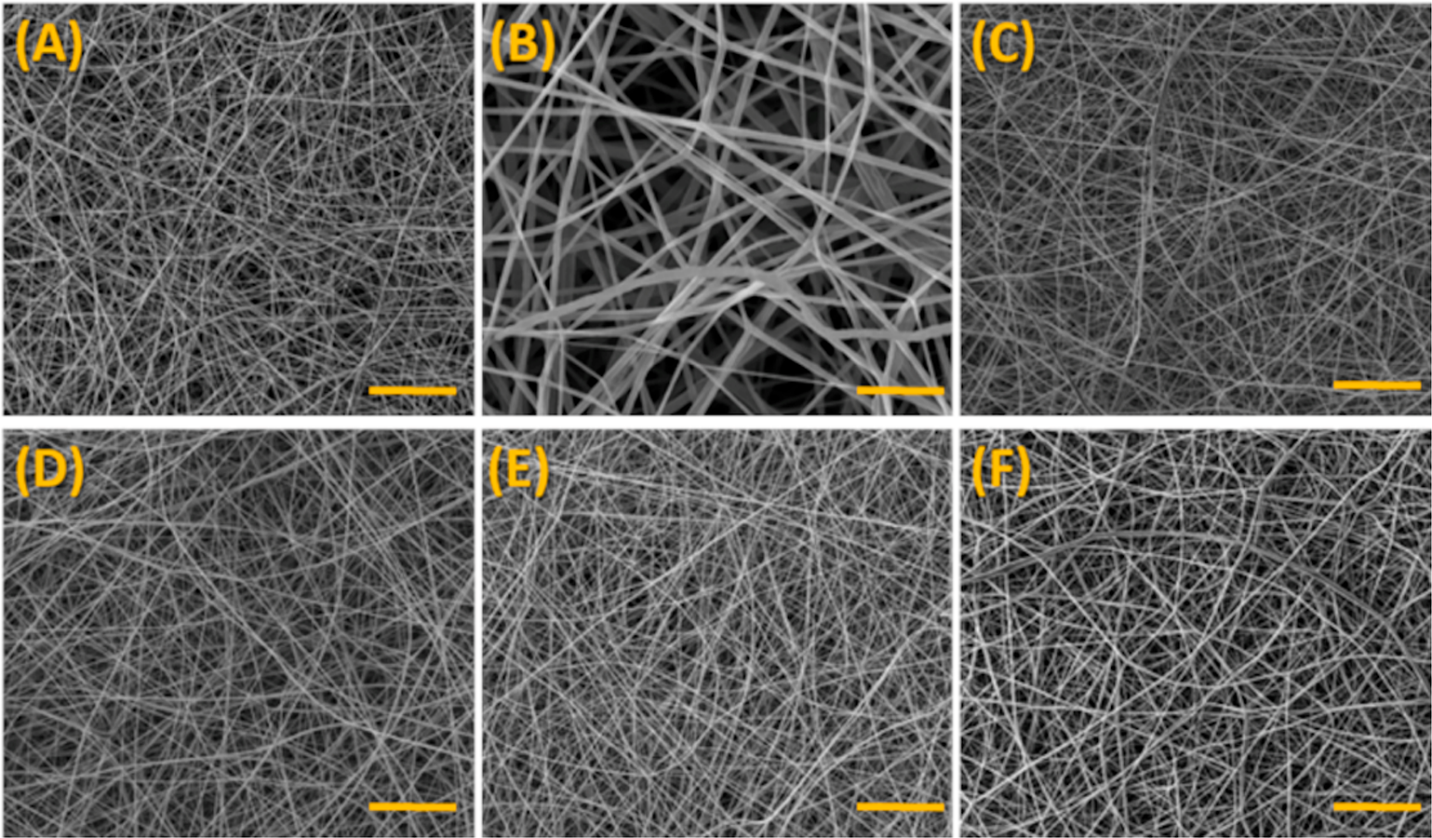
SEM micrographs of electrospun (A,B) PLA (magnifications of 1000× and 5000×, respectively; scale bar = 50 and 10 μm, respectively) and (C) PLA–CUR; (D) PLA–N(CH_3_)_2_; (E) PLA–N(C_2_H_5_)_2_; (F) PLA–N(Ph)_2_ (1000× magnification, scale bar = 50 μm).

Figure 6 shows the SEM micrographs for mats obtained from the electrospinning of PLA and PLA/curcuminoids solutions.

Under the electrospinning conditions used in this work, we demonstrated the successful production of homogeneous and defect-free fibrous mats for all groups considered (Figure 5). The average fiber diameters were calculated (at least 100 individual fibers) as follows: 728 ± 53, 730 ± 59, 776 ± 60, 773 ± 84, and 1012 ± 110 nm for electrospun mats from PLA, PLA–CUR, PLA–N(CH_3_)_2_, PLA–N(C_2_H_5_)_2_ and PLA–N(Ph)_2_, respectively. Thus, it is observed that the electrospinning process under the chosen conditions and parameters led mostly to ultrathin fibers (100 < diameters <1000 nm).

After dissolution, all PLA/curcuminoid solutions presented higher viscosities than pure PLA solution, which was clearly noticed to the naked eye. Regarding the average fiber diameters, although no clear trend can be assumed the increase in the standard deviations for PLA/curcuminoids and the greater increase in diameter for PLA–N(Ph)_2_ may be associated with the viscosity of the solutions. It is well-known that high viscosities impair the ejection of the polymer jet while interfering in the solvent evaporation. Assuming the expected greater hydrodynamic volume of N(Ph)_2_ in solution due to the presence of the aromatics rings, this curcuminoid may be responsible for the impairment of the entanglement of the polymers chains in some extension. As a result, the higher viscoelastic forces in the solution would contribute to a higher resistance to the axial stretching during electrospinning, which leads to thicker fibers.

**Figure 7:**
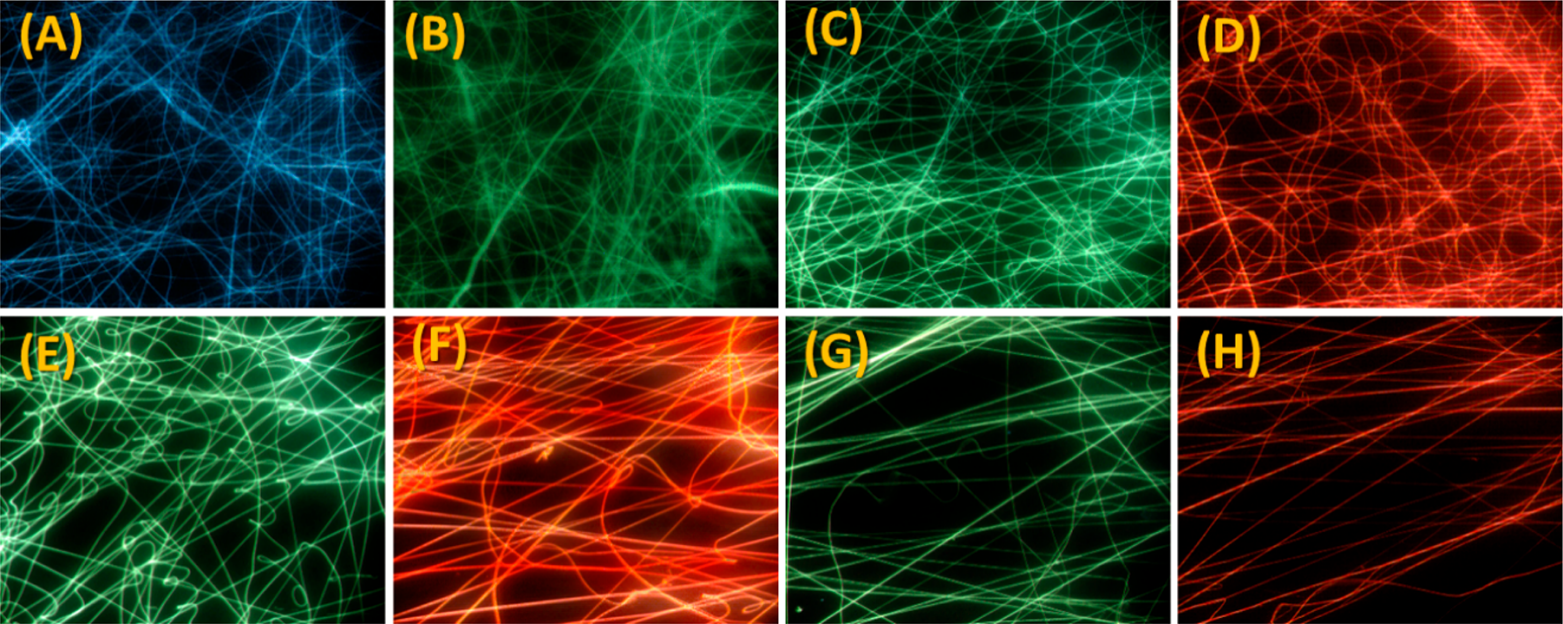
Optical fluorescence images obtained under different excitations: (A,B) PLA–CUR; (C,D) PLA–N(Ph)_2_; (E,F) PLA–N(CH_3_)_2_; (G,H) PLA–N(C_2_H5)_2_.

Figure 7 shows the images obtained by fluorescence microscopy from the excitation of the electrospun PLA/curcuminoids mats at different wavelengths. Different excitation wavelengths were considered. In this way, different excitation filters with narrowband passage windows were combined to excite the materials in different spectral regions: violet (385–400 nm), blue (475–490 nm), and green (545–565 nm).

As it can be observed in the optical fluorescence images, varied fluorescence emissions were obtained at different wavelengths, ranging from blue to red, under different excitation wavelengths. This result corroborates to the observed spectral results in the 3D Maps (Figure 5). Thus, it is proven that even in solid matrices, such as PLA, these curcuminoids present as fluorescence emission–excitation-dependent. It can be observed that for all electrospun PLA/curcuminoid mats, the fluorescence distribution was uniform throughout the fibrous web, which is indicative of a homogeneous distribution of the curcuminoids throughout the polymer matrix.

**Figure 8:**
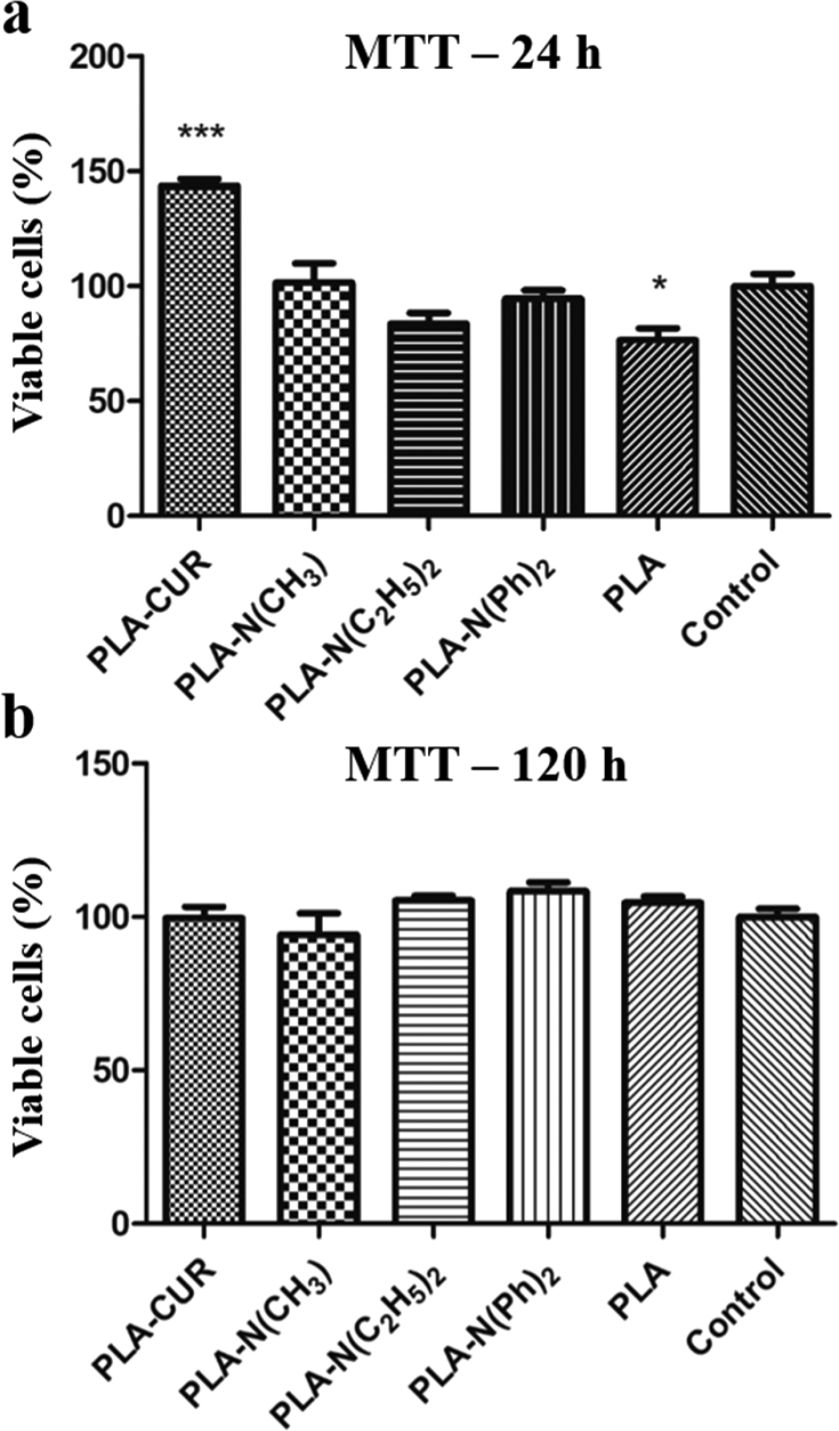
Cell viability of SH-SY5Y neuroblastoma in the presence of PLA, PLA–CUR, PLA–N (CH_3_)_2_, PLA–N(C_2_H_5_)_2_, and PLA–N(Ph)_2_ after (a) 24 h of incubation and (b) 5 days of incubation (120 h).

The results of the MTT analysis (Figure 8) did not show a significant decrease in cell viability after 24 h or 5 days (120 h) of incubation. After 24 h, a significant increase in viability was observed for the PLA–CUR fibers. This result corroborates with Zheng et al.,^53^ where the treatment of SH-SY5Y cells with curcumin (2.5–20 μmol L^–1^) for 24 h did not significantly affect cell viability. Yin et al.^54^ reported on the neuroprotection of curcumin against the oxidative stress induced by beta-amyloids, as determined by ELISA of SH-SY5Y cells transfected as plasmid APPswe, via activation of the PI3K/Akt/Nrf2 signaling pathway. The results indicated that the cytoprotection conferred by curcumin on SHs-SY5Y cells transfected with APPswe is mediated by its ability to regulate the balance between heme oxygenase 1 and 2 via the PI3K/Akt/Nrf2 intracellular signaling pathway.

Jaisin et al.^55^ found that pretreatment with curcumin improved cell viability and significantly reduced reactive oxygen species (ROS). Subsequent investigations revealed a reduction in p53 phosphorylation and a decrease in the Bax/Bcl-2 ratio, as measured by mRNA expression and protein level. According to the authors, these results indicated that curcumin protects dopaminergic neurons from 6-OHDA-induced toxicity by reducing ROS production and subsequently attenuating p53 phosphorylation and reducing Bax/Bcl-2 ratio.

**Figure 9:**
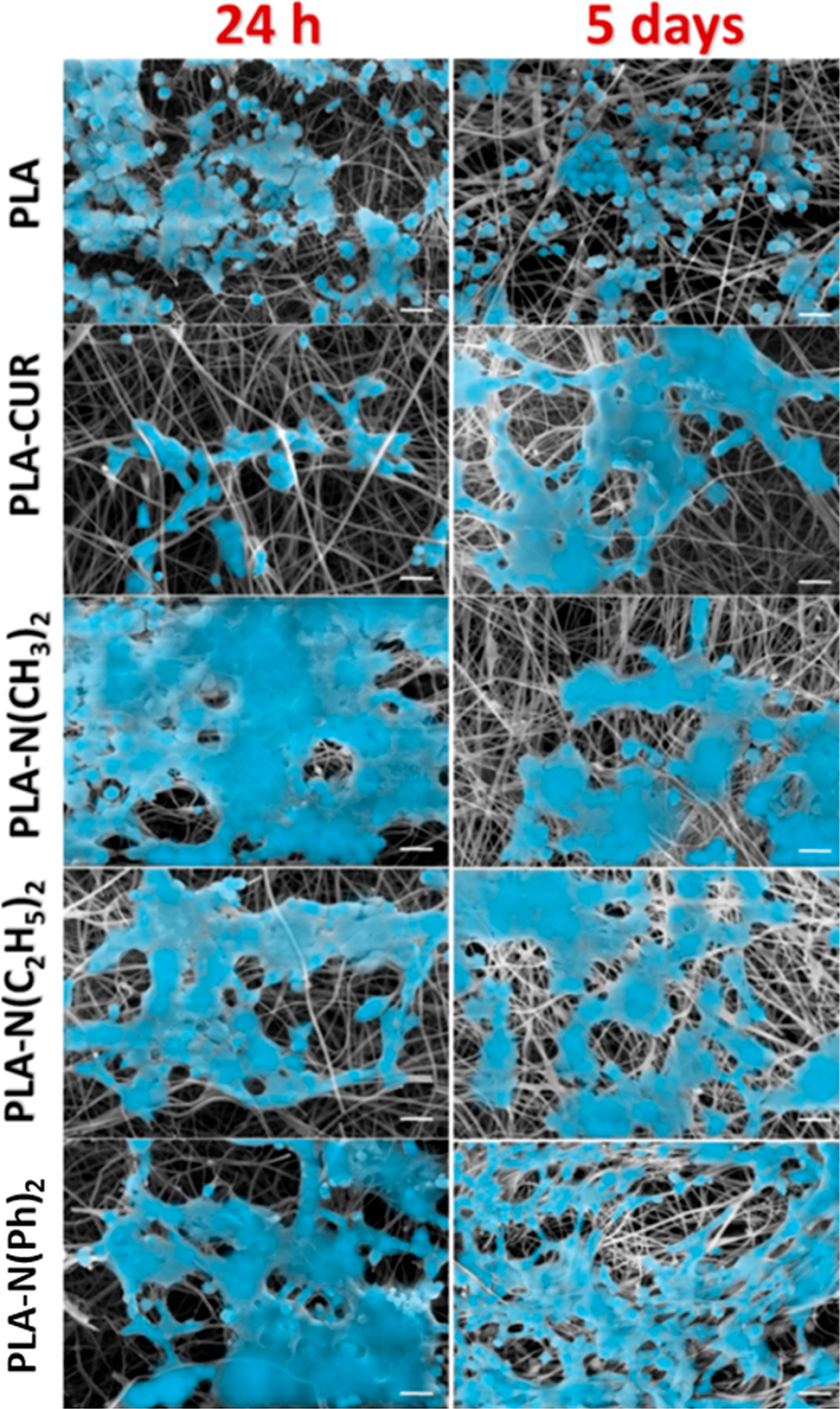
SEM micrographs revealing SH-SY5Y neuroblastoma adhesion and proliferation after 24 h and 5 days (120 h) onto the surface of electrospun PLA and PLA/curcuminoids (scale bar = 20 μm).

The SEM micrographs (Figure 9) initially corroborate with the cell viability results. Nevertheless, as we analyze the adhered cells, it is possible to observe that all curcuminoid-containing scaffolds except PLA/curcumin presented a visually enhanced cell adhesion and spread both after 24 h and 5 days, revealing the formation of cell monolayers. Conversely, electrospun PLA and PLA–CUR showed a more globular adhesion and too little cell proliferation after 24 h (PLA and PLA–CUR) and 5 days (PLA). While Figure 9 shows representative SEM micrographs of SH-SY5Y adhesion and proliferation, we were also able to quantify the cell spreading across the fibrous nanoscaffolds. After 5 days, the percentage of cells per area was about 35% for PLA, while it reached around 70% and 80% for PLA–N(C_2_H_5_)_2_ and PLA–N(Ph)_2_, respectively. It is well-known that when cells have a spherical shape, as observed in PLA (after 24 h and 5 days) and PLA/CUR (after 24 h), cell division decreases, therefore reducing cell spreading. While PLA–CUR showed an improvement in cell spreading per area (54%) after 5 days of culture, all other PLA/curcuminoids showed an even greater improvement. Similar results were reported in a study with electrospun nanofibers containing curcumin at 2, 5, and 10 wt %.^56^ Although the curcumin concentration was much higher than here (0.1 wt %), the authors have still shown by SEM micrographs that cells cultured for 24 h resulted in globular cells with evidence of lamellipodia and also spindle cells adhered to the scaffolds. After increasing the cultivation time to 2 and 3 days, the authors observed that the cultured cells were highly elongated and well spread on the fibrous surfaces. The cells formed intercellular junctions with adjacent cells and the cells also seemed to grow on the top of each other on the scaffolds, forming a multilayer of cells.

Mokhames et al.^57^ has just recently reported on the differentiation of induced pluripotent stem cells (iPSCs) into smooth muscle cells promoted by curcumin and how this condition was synergistically improved once curcumin is incorporated into nanofibers. In the presence of curcumin, protein adsorption, cell adhesion and cell viability increased significantly. Golchin et al.^58^ demonstrated that incorporating curcumin in chitosan/PVA/Carbopol/PCL fibers improved their biological behavior while increasing the mesenchymal stem cell viability.

The functionalization and or/modification of (nano)-fibers and the fibers’ surface has been a key strategy to improve cell adhesion, spreading, and migration.^27^ Since most biomaterials interact with cells through layers of proteins adsorbed onto their surfaces, one of the most important parameters of the cell–biomaterial interaction is the surface hydrophilicity, which allows for the covalent bonding of proteins. Herein, we evaluated the surface free energy according to the Owens method for all electrospun samples in terms of the dispersive and polar contributions via the contact angle measurements obtained from water and diiodomethane (Table 2).

**Table 2:** Contact Angle and Surface Energy Components of Electrospun PLA and PLA/Curcuminoids

	contact angle, θ (deg)		surface energy components (mN/m)		
sample	water	diiodomethane	dispersive	polar	total
PLA	89.7 ± 3.4	25.7 ± 1.5	45.9 ± 2.1	0.48 ± 0.12	46.4 ± 2.3
PLA–CUR	87.1 ± 2.4	26.4 ± 2.1	45.6 ± 1.8	0.88 ± 0.11	46.5 ± 1.9
PLA–N(CH_3_)_2_	72.0 ± 2.2	39.4 ± 1.1	39.9 ± 0.5	6.46 ± 0.14	46.4 ± 0.6
PLA–N(C_2_H_5_)_2_	68.0 ± 3.9	43.0 ± 2.0	38.1 ± 1.5	8.83 ± 0.21	46.9 ± 1.7
PLA–N(Ph)_2_	70.0 ± 1.6	40.5 ± 3.4	39.4 ± 2.1	7.50 ± 0.11	46.9 ± 2.2

The contact angle for electrospun PLA was around 90°, which reflects the hydrophobic nature of this polyester. Although the water contact angle has a strict correlation with the preparation method, for example, electrospinning, casting, extrusion, as well as processing conditions, different values are found in literature. Nevertheless, values for the water contact angle are always superior than 90° and up to 140° in some cases^59^ which confirm the hydrophobicity of this polyester. One can note that the inclusion of curcuminoids even at low contents led to a decrease in the water contact angles, which was more pronounced for PLA–N(CH_3_)_2_, PLA–N(C_2_H_5_)_2_, and PLA–N(Ph)_2_. For PLA–CUR, a more discrete decrease was observed, which would be actually the expected result due to the hydrophobic nature curcuminoids.

Although most of the curcuminoids are readily soluble in organic solvents, such as DMSO, acetone, and isopropanol, they are poorly soluble in water at neutral pH. The solubility is usually increased in alkaline conditions; however highly alkaline solutions lead to curcumin degradation, which generates 2,4-dioxo-5-hexanal, feruluic acid, feruloylmethane, and vanillin.^47^ The water insolubility of curcumin is associated with the large presence of phenyl rings along with the ability of hydrogen bond formation from the side groups, which lead to aggregation in water.^47,50^ Unexpectedly, we observed a decrease in the water contact angle for all samples containing low content of curcuminoids in the electrospun PLA matrix.

Hydrophobicity has often been described as a combination of low-energy surfaces and roughness.^60^ In nature, hierarchical roughness is found in many plants as a smart strategy for water uptake due to the presence of hierarchically structured surfaces.^61^ The surface energy is minimized while the wetting contact angle is reduced because air is trapped between nanostructured surfaces with a certain roughness.^62^ Compared to the most traditional structures, such as fibers and films, electrospun fibers exhibit superior properties for applications where hydrophilicity tailoring is desired, due to their porous nature, micro- and nanoscale diameters, large surface-to-volume ratio, and increased surface roughness.^63^ Overall, interfacial free energy correlates with surface deformation, which in turn is closely related to surface roughness and chemical composition. Thus, tailoring the properties of electrospun scaffolds, including surface roughness and porosity, affects the interfacial free energy and simultaneously alters the wettability of surfaces according to the Cassie and Baxter model.^64^ To date, several studies have indicated lower water contact angles with increasing fiber diameter,^65^ as also observed in our study (Figure 6). However, recent studies on the influence of surface geometry on the contact angle of electrospun scaffolds have shown that fiber diameter is not necessarily a driving parameter unless it is correlates with surface roughness.^66^

Regarding the surface free energy (γ), which comprises the sum of the dispersive and polar component, a similar value of approximately 47 mN/m was calculated for all electrospun samples, including PLA. It is well-known that the interfacial free energy determines the surface wetting characteristics, and therefore, the wall shear stress generated when the liquid comes into contact with the surface. Although the surface energy was the same for all electrospun samples, the dispersive and polar components had different contributions once the curcuminoids were added. As the values of the dispersive component decreased, the ones for the polar component increased from 0.48 up to 8.83 (18 times) for PLA–N(C_2_H_5_)_2_. The increase in the polar component is in agreement with the results of the water contact angle and cell adhesion and spreading, pointing out the higher hydrophilicity of the curcuminoid-based materials. Since the polar components of the surface attract the electric dipoles of water, this results in a reduction of the interfacial energy and, therefore, the water contact angle.

Curcumin has no fluorescence properties in aqueous media, although it becomes fluorescent in apolar/hydrophobic environments. Sneharani et al. reported on the decrease of the affinity of curcumin with the decrease in surface hydrophobicity of proteins.^67^ It is demonstrated that the aromatic amino acids of proteins establish hydrophobic interactions with the methoxyl phenyl group and the keto group of curcumin; the other major type of interaction with nonpolar amino acids of proteins is van der Waals.^68^ In sum, the interaction between curcuminoids and most materials is mainly dependent on the substrate’s surface hydrophobicity, while the absence of binding results in a rapid degradation.

PLA, as other polyhydroxy acids, has an intrinsic hydrophobic nature mainly due to a lack of sites for effective interaction with water molecules. Herein, we believe that the strong interactions between the PLA hydrophobic sites and the nonpolar groups from curcuminoids, for example, phenyl groups, and also the hydrophobic association between curcuminoids molecules may have been responsible for releasing hydrophilic sites from both curcuminoids and PLA in order to interact with water molecules. Thus, hydroxyl groups from curcuminoids, or even from the terminal chains of PLA, along with free carboxyl groups from both components would have been responsible for increasing the polar components at the surface, leading to an enhanced hydrophilicity. In literature, it is reported that this is the preferred interaction of curcuminoids with proteins and lipids, for example, via their movement into the hydrophobic groups of these macromolecules.^69^ Thus, binding to phospholipids in membranes and proteins and entrapment in hydrogels have been used as strategies to enhance the solubility and stability of curcuminoids in aqueous systems.^70^

While the hydrophobic associations between curcuminoid molecules and between curcuminoid and PLA are to be expected, the projection of the hydrophilic groups is not entirely obvious. Moreover, studies have reported a greater dependence of the advancing contact angle with the hydrophobic components on polymer surfaces.^71^ Here, the lower contact angles could also be due to the observed decrease in the contribution of the dispersive components due to their lower availability. Finally, as reported elsewhere and observed in the present work, the blue shifts in the emission maxima of all curcuminoids dispersed in PLA indicate an effective binding of these molecules to the hydrophobic polymer surface,^67^ which in turn would allow outwardly directed hydrophilic groups.

## Conclusion

In this investigation, we reported on the unexpected effect of tailoring the interfacial free energy of electrospun hydrophobic scaffolds by the incorporation of low contents of different curcuminoids. For this, we approached a straightforward method to produce homogeneous and defect-free photoluminescent PLA fibers, which were noncytotoxic, presented enhanced hydrophilicity, and greater cell adhesion and proliferation toward neuroblastomas. While all scaffolds presented very similar values of interfacial free energy, the presence of curcuminoids led to an increase in the contributions of the polar components along with the decrease in the values of the dispersive ones. This particular modulation of the surface free energy has been associated with the strong interactions between the hydrophobic sites and the nonpolar groups from curcuminoids, which may have been responsible for releasing polar/hydrophilic sites from both parts.

In this way, this study opens up a window of applications for future investigations, where PLA or other polyesters can be associated with low contents of curcuminoids in order to generate final materials with tailored properties at the biointerface combined with their well-known performance in protecting human neuroblastoma (SH-SY5Y) against oxidative damage to DNA. Since it was established that oxidative stress induced by hydrogen peroxide (H_2_O_2_) plays an important role in the etiology of several diseases, for example, Alzheimer, we will be able to evaluate the effectiveness and potential of these materials for applications as neuroprotective agents. In addition, further studies on the effect of UV and LED irradiations on these materials in terms of the phosphorescence of a singlet oxygen are currently being carried in our laboratories. Thus, in the future the potential of these photosensitive electrospun-based materials will be explored envisioning the application of photodynamic therapy for minimally invasive and toxic treatments.

## Supporting Information Available

^1^H NMR and ^13^C NMR spectra for curcumin and curcuminoid derivatives (PDF)
