# Supplementary material for: Curcuminoid-Tailored Interfacial Free Energy of Hydrophobic Fibers for Enhanced Biological Properties
Source: ACS Appl Mater Interfaces. 2021 May 24;13(21):24493–504. doi: 10.1021/acsami.1c05034 (PMC8289194; doi:10.1021/acsami.1c05034)
Supplement: Supplementary file 1 — am1c05034_si_001.pdf [file am1c05034_si_001.pdf]

Supporting Information

Curcuminoid-tailored interfacial free energy of hydrophobic fibers for enhanced biological properties

Wevernilson F. de Deus, Bruna. M. de França, Josué Sebastian B. Forero, Alessandro E. C. Granato, Henning Ulrich, Anelise C. O. C. Dória, Marcello M. Amaral, Adam Slabon*, Bruno V. M. Rodrigues*

AUTHOR INFORMATION

Wevernilson F. de Deus - Instituto Científico e Tecnológico, Universidade Brasil, Rua Carolina Fonseca 235, 08230-030, São Paulo - SP, Brazil

Bruna M. de França - Instituto de Química, Universidade Federal do Rio de Janeiro, Centro de Tecnologia, Bloco A, Cidade Universitária, 21941-909, Rio de Janeiro - RJ, Brazil.

Josué Sebastian B. Forero - Instituto de Química, Universidade Federal do Rio de Janeiro, Centro de Tecnologia, Bloco A, Cidade Universitária, 21941-909, Rio de Janeiro - RJ, Brazil.

Alessandro E. C. Granato - Departamento de Bioquímica, Instituto de Química, Universidade de São Paulo, CEP: 05508-000, São Paulo - SP, Brazil

Henning Ulrich - Departamento de Bioquímica, Instituto de Química, Universidade de São Paulo, CEP: 05508-000, São Paulo - SP, Brazil

Anelise C. O. C. Dória - Laboratório de Biotecnologia e Plasmas Elétricos, IP&D, Universidade do Vale do Paraíba, Av. Shishima Hifumi 2911, 12244-000, São José dos Campos, SP, Brazil

Marcello M. Amaral - Instituto Científico e Tecnológico, Universidade Brasil, Rua Carolina Fonseca 235, 08230-030, São Paulo - SP, Brazi

Adam Slabon - Department of Materials and Environmental Chemistry, Stockholm University, Svante Arrhenius väg 16 C, 106 91 Stockholm, Sweden; ORCID: 0000-0002-4452-1831

Email: adam.slabon@mmk.su.se

Bruno V M. Rodrigues - Department of Materials and Environmental Chemistry, Stockholm University, Svante Arrhenius väg 16 C, 106 91 Stockholm, Sweden; ORCID: 0000-0002-0130-8029

Email: bruno.manzolli@mmk.su.se

E-mail: adam.slabon@mmk.su.se
bruno.manzolli@mmk.su.se

Table of Content

Figure S1. ^{1}H NMR spectrum of curcumin
Figure S2. ^{13}C NMR spectrum of curcumin
Figure S3. ^{1}H NMR spectrum of Cur-N(Ph)$_2$
Figure S4. ^{1}H NMR spectrum of Cur-N(CH_3)$_2$
Figure S5. ^{1}H NMR spectrum of cur-N(C_2H_5)$_2$
Figure S6. ^{13}C NMR spectrum of cur-N(C_2H_5)$_2$

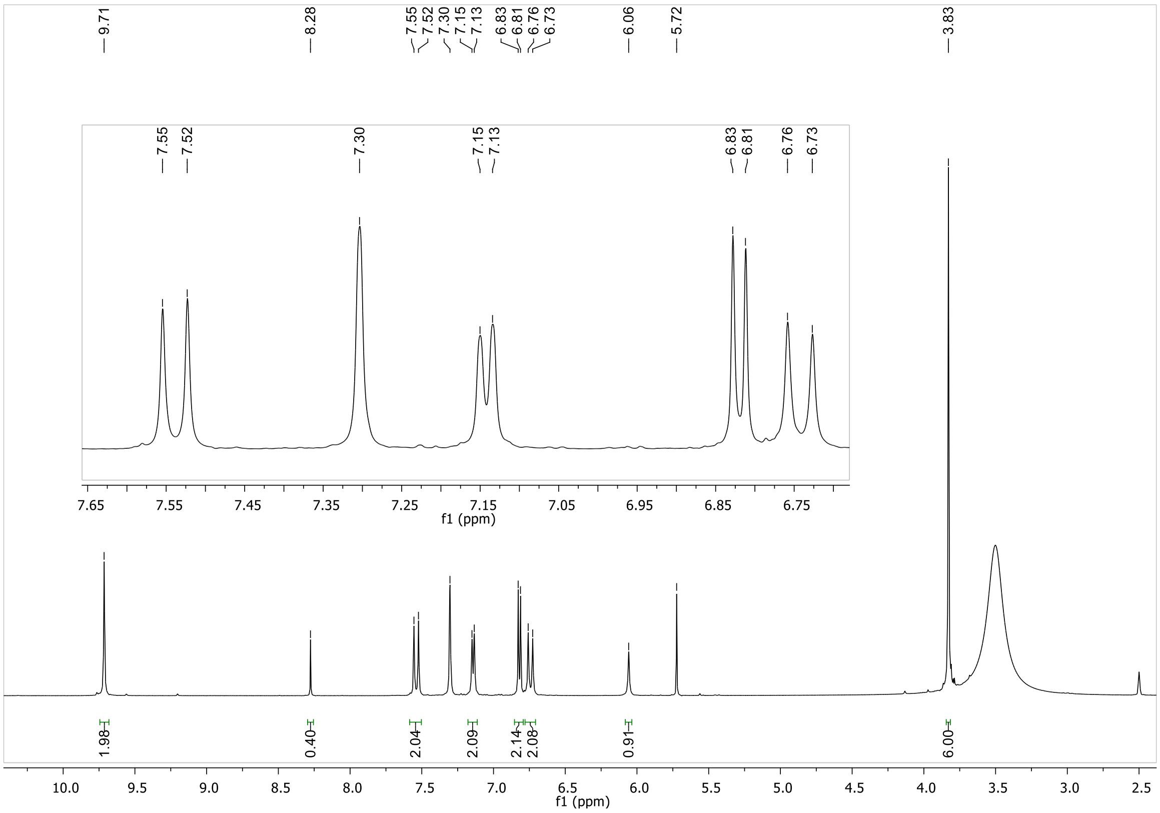

Figure S1. ^{1}H NMR spectrum of curcumin.

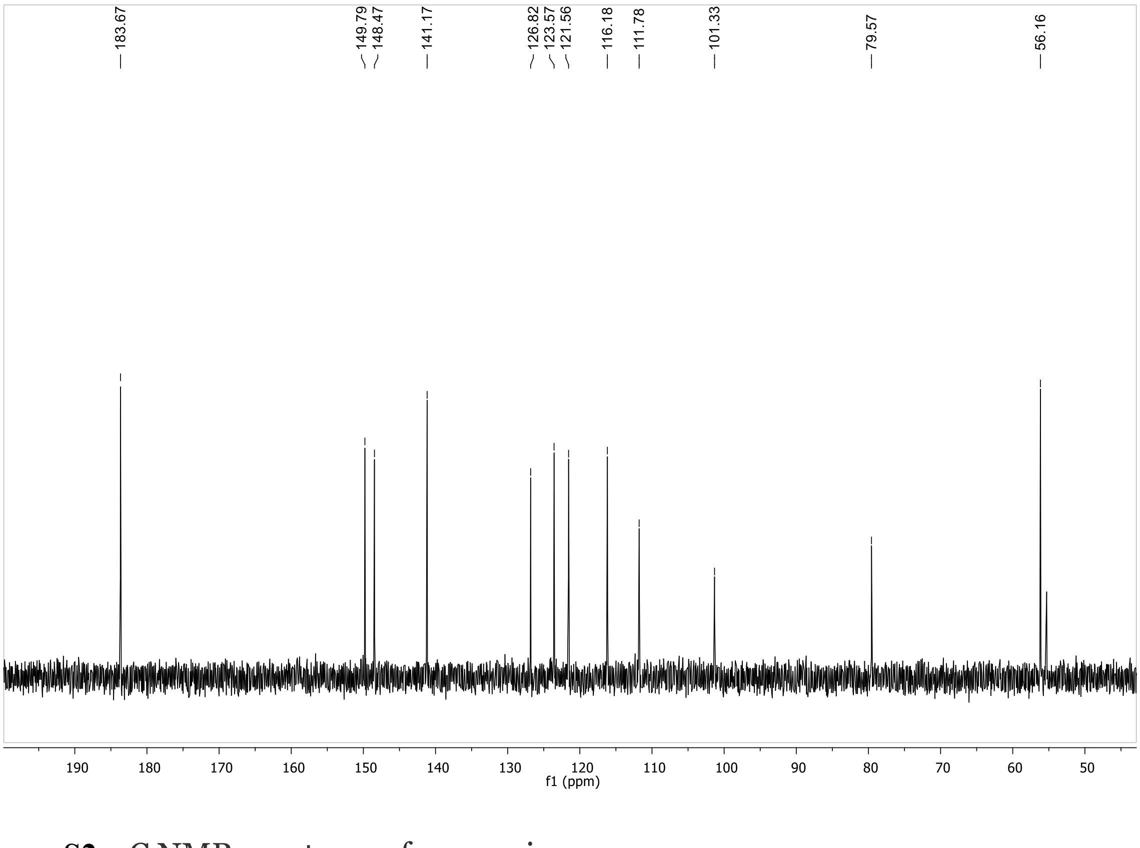

Figure S2. ^{13}C NMR spectrum of curcumin.

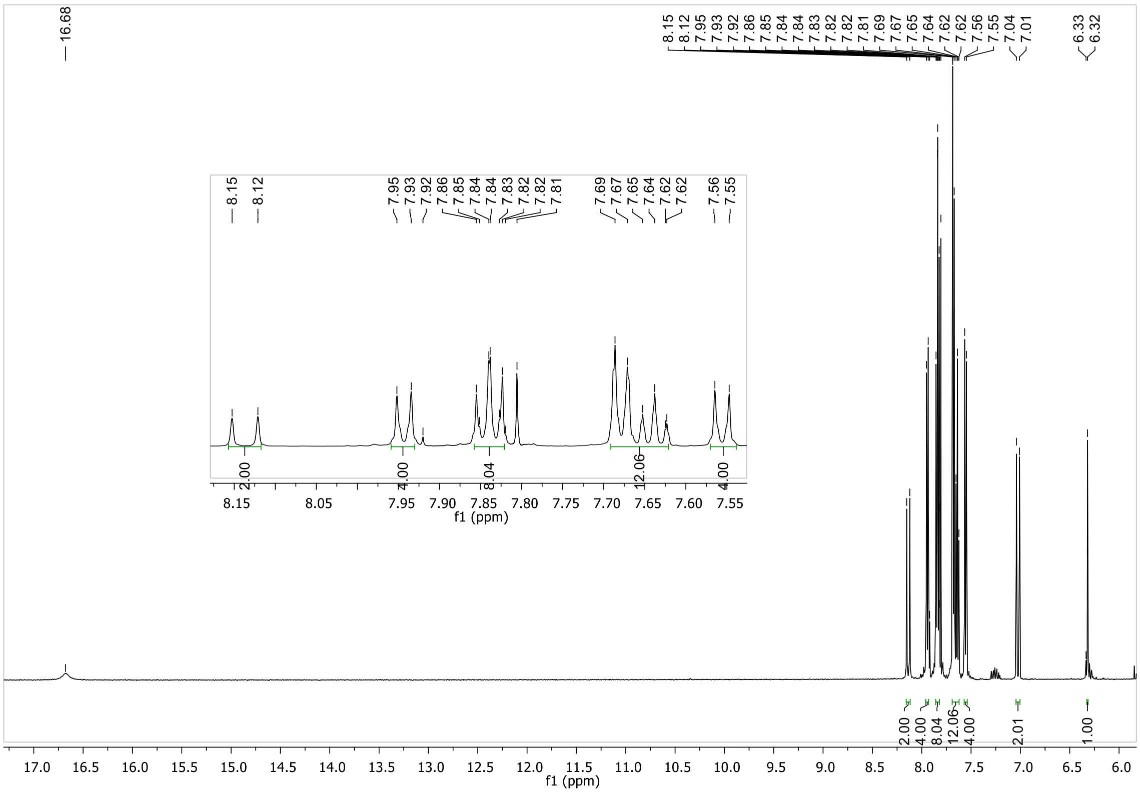

Figure S3. ^{1}H NMR spectrum of Cur-N(Ph)$_2$.

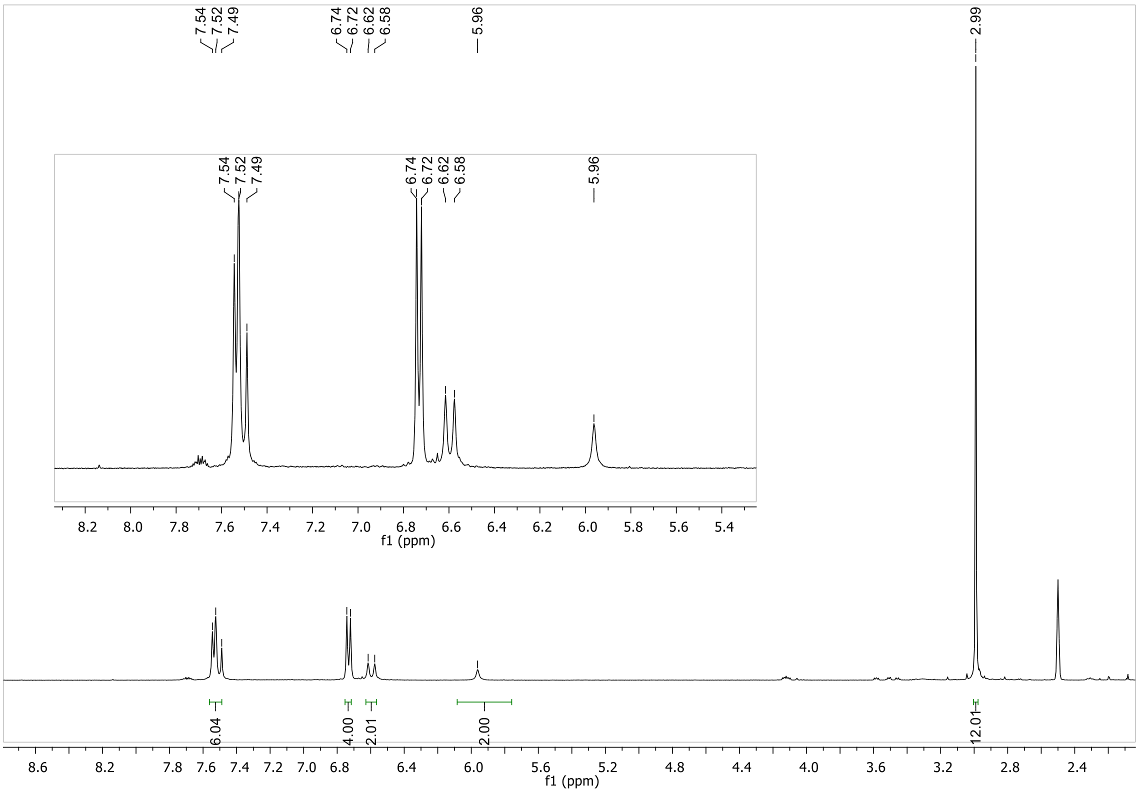

Figure S4. ^{1}H NMR spectrum of Cur-N(CH$_3$)$_2$.

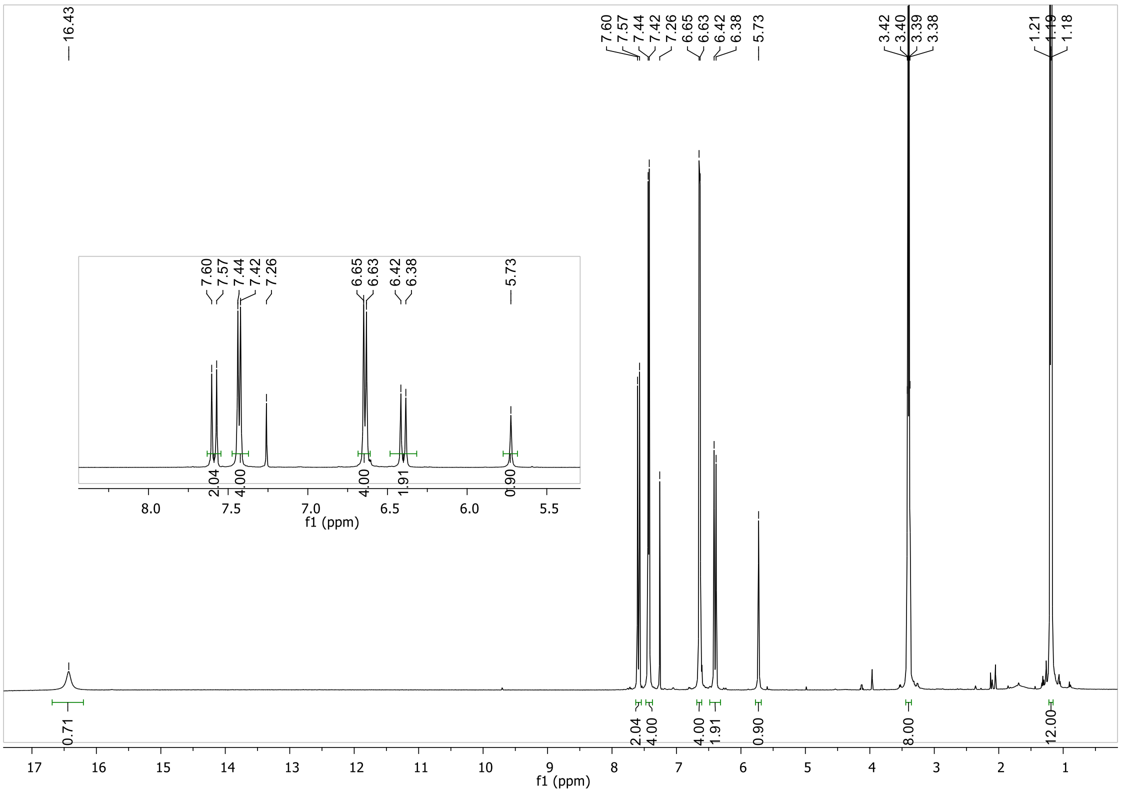

Figure S5. ^{1}H NMR spectrum of cur-$N(C_2H_5)_2$.

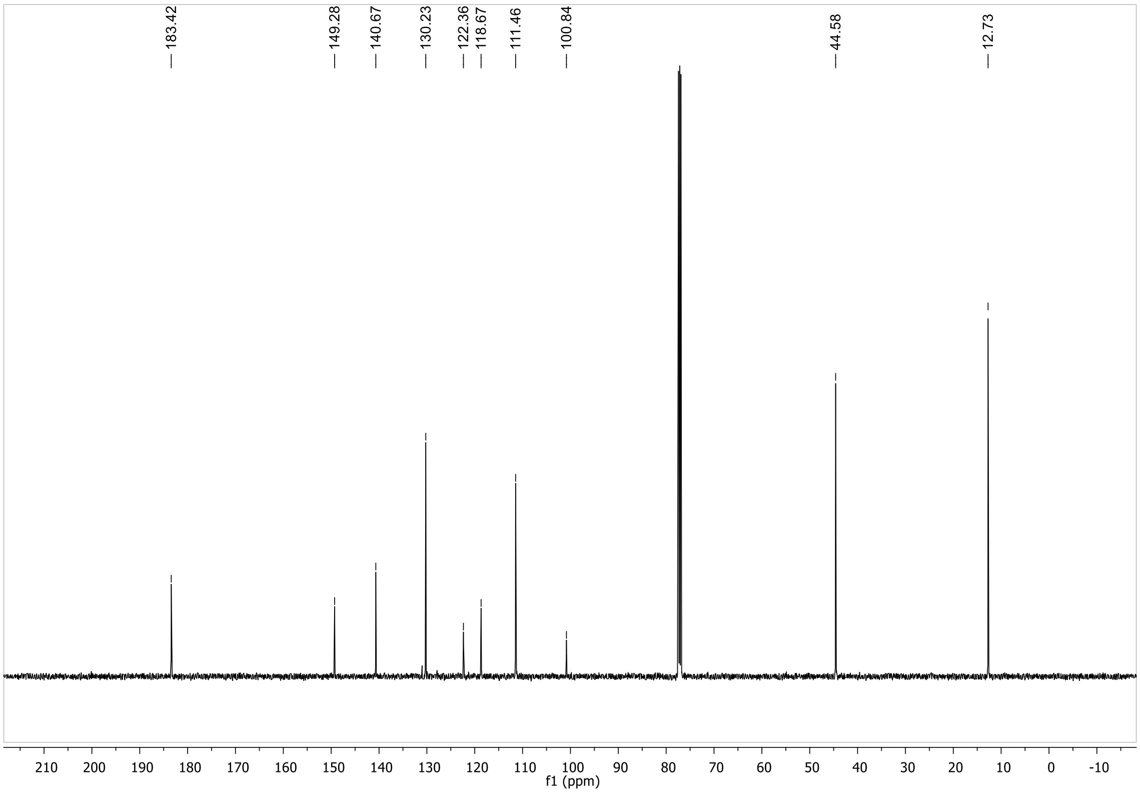

Figure S6. ^{13}C NMR spectrum of cur-$N(C_2H_5)_2$.
